# Supplementary material for: Feature identification in time-indexed model output
Source: PLoS One. 2019 Dec 4;14(12):e0225439. doi: 10.1371/journal.pone.0225439 (PMC6892550; doi:10.1371/journal.pone.0225439)
Supplement: Attachment — Submitted filename: Response to Reviewers.pdf [file pone.0225439.s002.pdf]

1 Code

If accepted we can upload well-anotated MATLAB codes for the production of the figures in the paper as example code for the method.

2 For All Reviewers

Thank you all for your very helpful and insightful reviews. We have extensively rewritten the manuscript with your comments in mind. I have included your original comments in plain text, along with my responses *in italics*

3 Reviewer 1

This paper describes a method to identify time points of interest in time series data via the reconstruction error of EOF. The method is sound and sufficient details are provided. The manuscript can be improved by the following points:

- The numbering of the sections throughout the manuscript is off. Please check to make sure they reflect the actual numbering. e.g. last paragraph of section 1, all numbers are off. *We checked this, and on our end all of the numbering appears correct. Perhaps there is an error in the submission system or something? Dr Stastna has experience reviewing for PLOS, and in one case there was a problem where the manuscript was correct but the reviewer copy had errors. Perhaps this is happening in this case. We have double checked the upload on our end, and everything is fine.*

- The structure of the manuscript can be reordered. The introduction of the datasets prior to the methods can be distracting since the focus of the manuscript is on the method. Also, the content of EOF can be reduced as this is not the innovation proposed by the authors. The authors should mention clearly what the contributions are compared to the existing literature. *We have merged the section on data sets with the results section, which should focus the manuscript on the method as you suggested. This also removes the need for the Figures introducing the data sets. We found that having the data sets directly before the results section was less readable than merging the introduction of each data set with its corresponding results. We think you will agree. Also, to clarify our contribution, we now explicitly outline the novelty of what we are presenting:*

  – The method is data driven, using a novel construction: a map of the EOF reconstruction errors as a function of time and the number of modes in the reconstruction. The interpretation of this EOF error map yields the identification of interesting times in each field in the data set for the cost of one Singular Value Decomposition (SVD) and one norm calculation per time output and choice of reconstruction.

This hopefully makes it clear that it is the error map interpretation that is the innovation. We have also rewritten the introduction, which now includes a comparison of our method to existing CFD analysis methods. The difference between our method and others is now clearly delineated. Finally, regarding the EOF background section, we understand wanting to reduce the presentation of the EOF as known material, but a review is necessary in order to set the notation and introduce all facts necessary for the presentation of the error map method. The error map method depends on a consideration of reconstructions, and so a full presentation is very helpful in setting up the subsequent arguments. It is true that some material could be omitted, but it is already short, and we have never seen this type of clear and thorough presentation of the SVD method as it relates to EOFs. It is standard to present very few details, but this acts as a barrier to entry for those not already familiar with the material, in the same way that a mathematical proof stripped of all clarifications, while still correct, becomes much more difficult to read. We have included all the details to eliminate any confusion about conventions or notation, as well as to highlight all mathematical facts relevant to the justification of the error map method. In order to increase readability we have left it largely unchanged.

- The results of this proposed method should be compared with some baseline methods in the literature. They should be applied to the same datasets and metrics of performance evaluation and comparison should included. *To our knowledge there are no comparable methods for time indexed model output in the literature. No other methods we know of identify periods of interest besides the gamma method we have just published Shaw et al. (2019), but that was designed for data sets consisting of time series sampling multiple physical fields. However, there are of course a wide variety of data analysis methods for CFD data sets. We have changed the introduction, including a review of a variety of other methods, in order to clearly delineate the difference between our method and others. The main difference is that our method is for identifying time periods worthy of further study. This further study would be in any form appropriate to the application, including any of these other methods. It is not a choice between our method and others, but that our method helps choose a time period to which these other methods would be applied. This is now clear in the text.*

4 Reviewer 2

This paper proposes a method for identifying time periods of interest in time-series data by using empirical orthogonal functions (EOF) or principal component analysis. Leveraging PCA to determine important modes in the specific application of fluid dynamics may be new but this reviewer is concerned with the novelty in the proposed method. PCA have been extensively used to determine important components of different signals. However, the current paper does not provide any comparison with the state-of-the-art algorithms in this area. Moreover, there are a few parameters such as D in this method that needs configuration

for which the authors are not providing a systematic solution; therefore, it is not clear in the paper how others can use this method to find important modes/components within a time-series signal. These two concerns makes the paper inappropriate to being published in the current form; Below are the detailed comments that need to be addressed:

- The authors need to provide comparison with the state-of-the-art. *We have provided more information in the introduction to address this.*
- Setting of parameter D should be clearly explained. *We have added a note clarifying the role of D in our derivations. In short, the parameter D is swept in the error map method, and so there is no need to choose a specific D. This is now clarified in the text.*
- The authors indicated that the proposed method "can minimize the cost of uptake and maximize the clarity of the presentation"; however, there is no results to support these claims. Please provide additional results/discussion about this. *It is not the method itself that "can minimize the cost of uptake and maximize the clarity of the presentation." It is the choice to base the method on the EOF which does this. Put another way, the fact that EOFs are widely known as a standard method implemented everywhere, many if not most people interested in the method are already familiar with many of the concepts, and can use existing implemented code in their favourite toolbox. Hopefully the changes in phrasing in the Introduction address this.*
- The authors provided a great deal of discussion in the introduction section about inability of the current methods regarding providing interpretable representations that carry physical meanings. However, there is no sign of interpretability in the proposed method. It seems that the proposed method does not explain physical phenomena either. *That is correct. Our method is applied before others. That is before the physical interpretation stage. The purpose is to find targets for other methods. As is now hopefully apparent through the rewrites, the focus is on the identification of time periods within model output which are worthy of further study. We now discuss many other methods commonly applied to CFD data sets, but as we now point out more clearly, our method is applied earlier in the analysis pipeline: after the CFD data set is produced, and before these other methods would be applied. We discussed EOF correlations with physical processes as a way to situate the fact that our method is instead based on the reconstruction perspective.*
- Most of the figures do not have axis labels and physical units. *As it says in the last sentence of the data sets section "As the focus here is on the data analysis method presented, all data sets throughout this manuscript are presented in terms of grid points, time output number, and numeric field values."*
- Most of the formulation provided in section 3 are the typical equations used in PCA/EOF; the authors need to clarify which parts are novel, where are the gaps that they are addressing, and where and how they have improved the current formulation. *The first two sections of the methods section are indeed background material, in order to firmly*

set the notation used and facts needed. The EOF error map method is the novel construction. We have added clarifications for these points.

- In the text figure 7 has referred before figure 5. Fix the ordering. *Figure 7 is the scree plot, it is mentioned earlier as an example, in case a reader is unfamiliar with them. However it still appears later as the data sets are introduced (including Fig 5) before the results of the SVD are presented (including Fig 7). The text now makes this clear.*

- For the results section show how the ”time period of interest” is detected with the proposed method. *The examples in the results section outline that the time period of interest is indicated by the presence of changes in the error map over time.*

- Provide a few examples about the application of determining ”time period of interest”, especially, in applications other than fluid dynamics. *We have purposely narrowed the scope to only include CFD data sets. However, we are clear that it is an EOF based data-centric method, which means it is appropriate for use with any data set for which an EOF decomposition is appropriate.*

5 Reviewer 3

In this study, the authors gave an analysis on Feature identification in time-indexed model output. The introduction provides a good, generalized background of the topic. The following points should be clarified:

- The motivations for this study need to be made clearer. *The introduction was rewritten with this in mind, and now includes examples and a variety of clarifications on the method's motivation and purpose. Hopefully the motivation is more clear now.*

- However, to make the motivation clearer and to differentiate the paper some more from other applied papers, the author may wish to provide examples of some of the applications. *Again the introduction clarifies this, including a discussion of the method's use in large coupled models. Applications of the method are included in the Results section.*

- The authors must be explained how their method is novel and on what basis (justifications). *It is now clearly identified that the error maps are the novel portion of the work. The methods section includes as a primary justification the thought experiment beginning in paragraph 2 of the EOF error map section, and the tutorial figure showing a schematic of small, medium, and large variance processes. There is no clear way to proceed with a more rigorous derivation of the error map without imposing some kind of mathematical assumption on the form of the SVD. As it is a data driven method, and data can come from an extremely wide variety of sources, any such assumptions would severely limit the scope of the method, which is the opposite of our intent.*

- The authors did not mention the importance of Feature identification in time-indexed. *There is now additional material in the introduction to make this clear.*

- Also the authors are advised to update the manuscript before final submission as it contains some typos and grammatical errors. *We have done so, hopefully it is in better condition, our apologies.*

- The authors claim that their method is easily implemented and computationally inexpensive. It should be explained in detail for the interest of reader make it more interesting. *This comment was in reference to the fact that our method is based on carrying out the SVD, which is itself implemented in every major toolbox, and that those toolboxes implement the SVD in ways that make it computationally inexpensive.*

- My recommendation is a Major revision. *The manuscript has been thoroughly reworked. Hopefully the changes we have made will be satisfactory.*

6 References

References

Justin Shaw, Marek Stastna, Aaron Coutino, Ryan K. Walter, and Eduard Reinhardt. Feature identification in time series data sets. *Heliyon*, 5, 2019. ISSN 24058440. doi: 10.1016/j.heliyon.2019.e01708.
